# Supplementary material for: Programmable Electrofluidics for Ionic Liquid Based Neuromorphic Platform
Source: Micromachines (Basel). 2019 Jul 17;10(7):478. doi: 10.3390/mi10070478 (PMC6680446; doi:10.3390/mi10070478)
Supplement: Supplementary file 1 [file micromachines-10-00478-s001.zip › Supplemental Information - MM.pdf]

micromachines

MDPI

Supplementary Materials: Programmable Electrofluidics for Ionic Liquid Based Neuromorphic Platform

Walker L. Boldman, Cheng Zhang, Thomas Z. Ward, Dayrl P. Briggs, Bernadeta R. Srijanto, Philip Brisk and Philip D. Rack

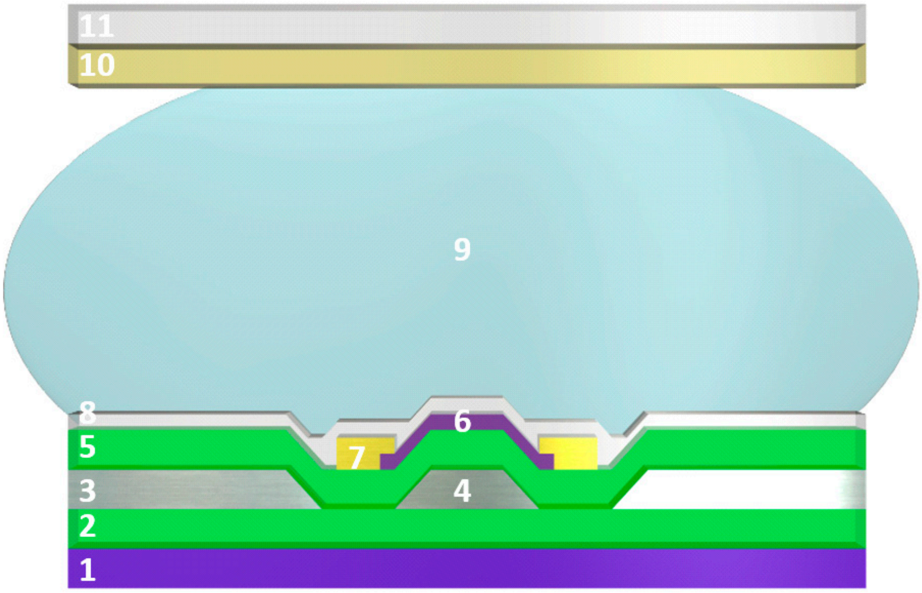

Figure S1: Labeling of 2D cross-section of TFT Active Layer. (1) Silicon substrate, (2) SiO_2 insulator, (3) EW electrode, (4) TFT back gate, (5) gate dielectric and EW insulator, (6) IGZO active layer, (7) source/drain electrodes, (8) hydrophobic layer, (9) ionic liquid, (10) top ITO gate, (11) glass slide.

Information S1: Fabrication of Transistor Array Device

The aIGZO thin film transistor array was integrated co-planar with a simple electrowetting array and both were fabricated on a silicon substrate with a 500 nm silicon oxide insulating layer. A 150 nm thick chromium back gate and co-planar electrowetting electrode was sputter deposited onto a 500 nm SiO_2 coated silicon wafer and then was lithographically patterned and wet etched with a Cr wet etch solution (9%$(NH_4)2Ce(NO_4)_6$ + 6%$(HClO_4)$ + H_2O). A 100 nm SiO_2 gate dielectric and electrowetting insulator was deposited via plasma enhanced chemical vapor deposition (PECVD). The 50 nm a-IGZO active layer was rf magnetron sputter deposited at room temperature using an In_2O_3:Ga_2O_3:ZnO mixed and pressed powder target with a 1:1:1 mol % ratio, and patterned by lift-off. The rf sputtering power was 80 W and the sputtering pressure was 5 mTorr with a 10:1 mixing ratio of argon:oxygen at a fixed total flow rate of 25 SCCM. 10/80 nm Ti/Au source and drain electrodes were deposited by e-beam evaporation and patterned by lift-off. To achieve eletrowetting of the IL, ~400 nm of TeflonAF 1600 was spin coated at 900 rpm for 60 s, and baked at 115 °C for 15 min, 165 °C for 15 min, and then 315 °C for 30 min.

Video S1: Full Device Concept Video: See video File

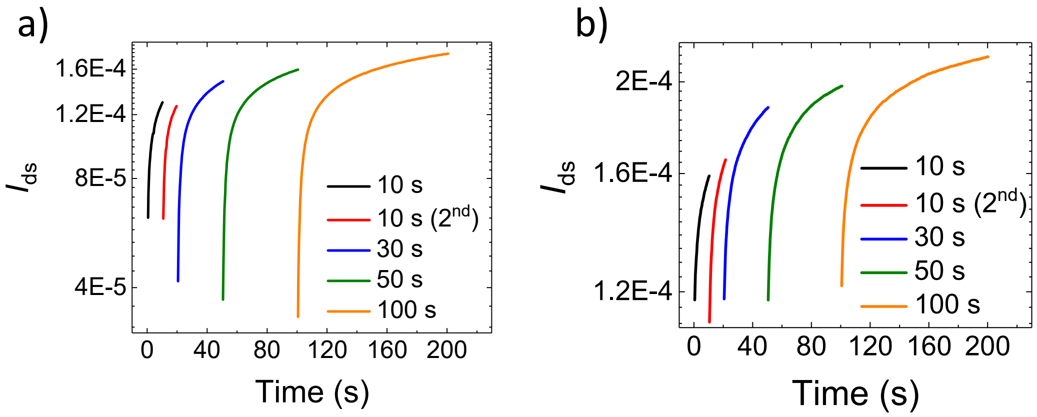

Figure S2: I_{ds} versus Top Gate Bias Time. I_{ds} as a function of cumulative bias time for (**a**) LiClO–PEO and (**b**) DEME.

Information S2a: Electrowetting Setup

To achieve electrowetting, a top plate was fabricated by DC sputtering 100 nm of transparent indium tin oxide (ITO) onto a glass slide and was suspended over the EWOD platform by ~80 µm spacers (see figure on EW setup).

A voltage generating program was developed using LabView software, using a data acquisition card (DAQ) to send signals to solid state relays (Vishay Semiconductors LH1512BB). Two external power supplies (Keithley 2400 Sourcemeter) and 8 relays were used. 5 V signals generated by the DAQ card are sent to the relays, which then sent higher voltages to the EW electrodes. Electrical connections between the EW electrodes and the relays were made using a test clip with spring loaded pins. For EW, an insulating silicone oil (Dow Corning OS-30) was filled between the EW device and the ITO top plate and the DEME IL was inserted into the oil where limited solubility exists between the oil and DEME.

Information S2b: Analysis of Electrowetting Actuation

Movement speed of the DEME and a yellow ink (Cab-O-JET 270 Yellow Colorant) were captured on a DSLR video camera, and the time needed for the IL droplet to move across one pad (2 mm) was recorded. To ensure that the results were repeatable and reliable, each droplet was moved left and right two times (four movements), with the time recorded in Figure 5d being the average of these measurements.

Video S2: Ionic Liquid Channel Formation: See Video File

Video S3: Ionic Liquid Actuation: See Video File
